# A call for a shared future vision for Planetary and One Health Literacy

**DOI:** 10.1093/heapro/daaf200

**Published:** 2025-11-26

**Authors:** Carmen Jochem, Gerardine Doyle, Kristine Sørensen, Ilona Kickbusch, Simon Rüegg, Saskia Maria De Gani

**Affiliations:** Chair of Planetary & Public Health, University of Bayreuth, Universitaetsstraße 30, 95447 Bayreuth, Germany; Department of Epidemiology and Preventive Medicine, University of Regensburg, Franz-Josef-Strauß-Allee 11, 93053 Regensburg, Germany; College of Business, University College Dublin, Belfield, Dublin 4, Ireland; UCD Geary Institute for Public Policy, University College Dublin, Belfield, Dublin 4, Ireland; Global Health Literacy Academy, Borresøvej 26, 8240 Risskov, Denmark; Global Health Center, Geneva Graduate Institute, Chemin Eugène-Rigot 2A, 1211 Geneva, Switzerland; Section of Veterinary Epidemiology, University of Zurich, Winterthurerstrasse 270, 8057 Switzerland; Network for Ecohealth and One Health, Seraphin De Grootstraat 11, 2100 Antwerp, Belgium; Center for Health Literacy, Careum Foundation, Pestalozzistrasse 3, Zurich 8032, Switzerland; Careum School of Health, Kalaidos University of Applied Sciences, Gloriastrasse 18a, Zurich 8006, Switzerland

**Keywords:** health literacy, planetary health, One Health

## Abstract

Global health is increasingly shaped by interlinked crises such as climate change, biodiversity loss, pollution, and social inequalities, all of which undermine the determinants of health. At the same time, the digital revolution and geopolitical instability amplify misinformation and inequities. Health literacy has been recognized by the WHO Global Health Strategy as a key pillar of resilient health systems, while the Lancet One Health Commission highlights the urgent need for shared competencies across human, animal, and environmental health. Against this backdrop, the concepts of Planetary Health Literacy and One Health Literacy provide complementary frameworks to extend health literacy into ecological systems and the interconnected health of humans, animals, and other species. Planetary Health Literacy emphasizes sustainability and ecological boundaries, whereas One Health Literacy focuses on interspecies risks such as zoonoses and antimicrobial resistance. Together, they provide a powerful approach for fostering competencies that enable individuals, communities, professionals, and policy-makers to critically appraise information, make sustainable and health-promoting decisions, and act across human and ecological systems. This article calls for a shared vision of Planetary and One Health Literacy to guide health promotion, education, and policy. Key action priorities include embedding these literacies across all levels of education and professional training; developing and validating indicators for measurement; incorporating them into public health policies and climate-health frameworks; fostering cross-sectoral collaboration; and including indigenous and traditional knowledge. By investing in Planetary and One Health Literacy, governments and institutions can empower societies to adopt healthier, more sustainable behaviours, build climate-resilient health systems, and advance a systemic response to today’s polycrisis.

Contribution to Health Promotion

- This article shows why Planetary and One Health Literacy are essential for addressing today’s environmental, ecological, health, and social crises.
- It explains how linking sustainability and health literacy helps people including professionals make better decisions for health promotion and sustainability in everyday life and policy.
- It provides practical policy options, examples of good practice, and tools for measuring progress in health and sustainability literacy.
- The shared future vision of Planetary and One Health Literacy may support fairer, stronger, and more resilient communities by promoting health literacy that includes people, animals, and the environment.

## A CALL FOR A SHARED FUTURE VISION FOR PLANETARY AND ONE HEALTH LITERACY

The recently published WHO Global Health Strategy 2025–2028 emphasizes health literacy as a key pillar for strengthening equity-focused, resilient health systems worldwide in a turbulent world (World Health Organization 2025). The Lancet One Health Commission also calls for ‘the adoption and implementation of a standardised framework for core One Health competencies’ (Winkler *et al*. 2025). This call gains urgency in light of the current polycrisis: Global environmental and ecological crises not only coincide but also actively interact with the digital revolution. This transformation shapes material footprints through energy use, resource extraction, and e-waste, influences public understanding and collective action through digital information ecosystems, and reinforces commercial determinants that affect consumption patterns and policy priorities. These developments unfold within geopolitical changes and a transforming global governance landscape, where economic and technological dependencies are reshaping power dynamics between states and regions. Fragmented multilateral co-operation and competing geopolitical interests often hinder coordinated responses to planetary crises. Combined with the spread of mis- and disinformation and widening social and health inequities, these interlinked dynamics amplify vulnerabilities, contributing to rising burdens of disease, food insecurity, social inequality, and injustice (United Nations 2024). To effectively address these intertwined problems and existing implementation challenges—namely the persistent governance and institutional barriers that delay recognition and coordinated responses to complex health and environmental threats—there is a great need for a shared vision of a better future. With this comment, we discuss the role and the potential of a shared vision for Planetary and One Health Literacy.

The concepts of Planetary and One Health reflect the close connection between human health, animal health, and the health of other ecosystems, even beyond the planet (World Health Organization 2021b). In a holistic approach, these integrative health concepts address the cobenefits of health-promoting and ecologically sustainable behaviour and competencies to adopt, e.g. a healthy and sustainable diet and create conducive living environments for human health and flourishing natural systems (World Health Organization 2021a). However, this broad understanding of health is not yet adequately reflected in the current understanding of health literacy. Health literacy represents ‘the personal knowledge and competencies that accumulate through daily activities, social interactions and across generations’ (World Health Organization 2021b). It includes the ability for ‘critical judgement of health information and resources, as well as the ability to interact and express personal and societal needs for promoting health’ (World Health Organization 2021b). By pursuing a public health approach, health literacy may contribute to enhanced healthcare, disease prevention, and health promotion (Sørensen *et al*. 2012).

The Ottawa Charter not only laid the foundation for health promotion, but it also emphasized the relevance of supportive environments for human health (Ottawa Charter for Health Promotion 1986). Accordingly, not only social but also environmental and ecological determinants are among the determinants of health (Myers *et al*. 2025). Yet, in times of complex challenges such as planetary crisis, wars, and digital mis- and disinformation, these determinants of health are deteriorating (Kickbusch and Holly 2023), and further competencies such as Planetary and One Health Literacy are needed to deal with such complex challenges to establish and maintain health and well-being of all—including the planet itself. Planetary and One Health Literacy reflect the expanded understanding of health and the interconnectedness of people, nonhuman life, and the environment in the context of everyday decisions (Jochem *et al*. 2023, Blankart *et al*. 2024).

Planetary and One Health Literacy build on established understandings of health literacy, extending it into ecological and multispecies domains. We use the definitions of Planetary Health Literacy as ‘the knowledge and competencies of accessing, understanding, appraising, and applying information to make judgements and decisions regarding planetary health, across societies and for health-promoting, sustainable, and transformative actions. Planetary health literate individuals and societies are enabled to sustain and promote their own health, population health, and the planet's health. They are able to adopt a more holistic understanding of their health embedded in natural systems they are living in. Based on their knowledge and attitude, they make decisions that reflect and foster the interconnectedness of human health and well-being with the state of the natural systems and related areas of nature-society interactions’ (Jochem *et al*. 2023). One Health Literacy refers to ‘the knowledge, motivation and competencies to access, understand, appraise and apply all relevant information that are related to One Health in order to make judgments, take decisions and actions in everyday life concerning healthcare, disease prevention and health promotion to sustainably balance the health and quality of life of us humans, the animals and the environment’ (Blankart *et al*. 2024).

While Planetary Health Literacy emphasizes ecological boundaries and sustainability, One Health Literacy focuses on interspecies health risks, especially zoonoses and antimicrobial resistance. Despite their distinct emphases, Planetary Health Literacy and One Health Literacy are deeply interconnected and mutually reinforcing. Both extend the concept of health literacy beyond the human individual towards shared systems of health that link people, animals, and ecosystems. One Health Literacy contributes operational and governance-oriented competencies to address concrete interspecies and environmental health threats—such as zoonotic diseases or antimicrobial resistance—through coordinated multisectoral action. Planetary Health Literacy, in turn, situates these actions within broader ecological and societal systems, highlighting sustainability, justice, and the preservation of planetary boundaries as essential conditions for the health of current and future generations. Together, they form an integrative health literacy framework that combines health promotion with transformative sustainability goals, bridging the micro-level of individual action with the macro-level of systemic change.

Both forms of health literacy are acquired through continuous learning across multiple contexts—formal education, professional training, community engagement, and participatory policy processes. Individuals can strengthen their competencies by engaging with evidence-based environmental and health information, adopting reflective and collaborative learning approaches, and applying this knowledge in personal, professional, and civic decisions. Organizations, including educational institutions, public health agencies, and civil society actors, play a key role in enabling these learning environments by integrating Planetary and One Health perspectives into curricula, health communication, and governance structures. Together, Planetary Health Literacy and One Health Literacy offer complementary lenses for fostering global health, health promotion, and sustainability in the Anthropocene.

Combining these two concepts into an integrated understanding of Planetary and One Health Literacy offers a powerful framework that bridges human health with ecological sustainability with interspecies health interdependence. This is supported by the call for collaboration between Planetary Health and One Health as the underlying concepts (Castañeda *et al*. 2023). A synthesis of Planetary and One Health Literacy can support a systemic approach that could enable individuals, professionals, and policy-makers to understand how their behaviours impact not only their own health but also the health of animals and other ecosystems. It would promote the development of cross-sectoral competencies, such as the ability to critically evaluate health information through a human, ecological, and interspecies lens and to take action that supports cobenefits across environmental, human, and animal health systems.

The benefit of this integrative approach lies in its potential to build a bridge between a so-far siloed thinking in education, policy, and health(care) practice. It allows for aligned messaging and communication, shared tools and assessments, and coordinated interventions across domains traditionally seen as separate. However, combining the two concepts also presents challenges: their disciplinary roots differ (veterinary/public health vs. environmental health/sustainability), and with a newly emerging definition, there is a risk of conceptual blurring, dilution, or mutually exclusive competition. Thus, operationalizing a combined health literacy framework of Planetary and One Health Literacy requires careful and continuous negotiation of terminology, consensus on core competencies, and inclusive multi-stakeholder engagement to ensure relevance across diverse sociocultural and professional contexts and time. To stimulate the joint development of the new field of Planetary and One Health Literacy, we propose a first working definition: ‘Planetary and One Health Literacy can be defined as the knowledge, motivation, and competencies to access, understand, appraise, and apply information in order to make informed decisions and take actions that take into account the interconnectedness of the health of humans, animals, and ecosystems and thus promote health across generations and species within the limits of the planet.’

As researchers and health literacy advocates, we call for the adoption of a more comprehensive understanding of health—as integrated in the Ottawa Charter and the Geneva Charter—which focuses on ‘Well-being for All’, Health Literacy and Planetary and One Health Literacy concepts to reflect the strong interconnectedness of human health and other ecosystems across all scales, to sustain and strengthen all.

To develop a shared vision for Planetary and One Health Literacy, we suggest the following pragmatic and stepwise action priorities: first, we recommend considering Planetary and One Health Literacy as a joint, cross-sectoral goal that links awareness raising with the development of concrete competencies to promote health at all scales. Implementation should begin within existing structures, e.g. through integrating Planetary and One Health Literacy into health professional education, teacher training, and policy leadership programmes, before scaling to wider educational levels such as schools and community education. Initial pilot initiatives could focus on continuing education for the health workforce and climate-health policy actors, who may act as change agents and multipliers and where immediate cobenefits for health and sustainability can be demonstrated.

Second, there is a need for targeted and applied research to operationalize and measure Planetary and One Health Literacy. This includes, e.g. research to codevelop context-specific indicators and tools that quantify Planetary and One Health Literacy and explore their links to measurable outcomes such as health-promoting behaviour, sustainable lifestyles, and resilience. This requires participation of different target groups, including health professionals, policy-makers, lay people, and teachers, to better understand the implications of different ways of knowing, power dynamics, justice, and equity around Planetary and One Health Literacy (Jochem *et al*. 2023). Specifically, there is a critical need to include indigenous and traditional knowledges and adopt ‘Two Eyed Seeing’ in the operationalization (Bartlett *et al*. 2012). Governments need to play an active role in supporting the development and implementation of such indicators. A preliminary roadmap for implementation with a candidate metric for Planetary and One Health Literacy may include (i) knowledge of environmental health determinants and of cobenefits for health, environment, and sustainability; (ii) critical appraisal of ecological consequences of health-related decisions; and (iii) behavioural intention to support cobeneficial health and environmental outcomes. Specific research questions include, e.g.: How can individual and organizational Planetary and One Health Literacy be measured reliably across countries? Which educational and communication interventions most effectively enhance Planetary and One Health Literacy? We suggest that the WHO and its Member States pilot-test such composite indicators within regional and national health literacy surveys or as part of the Global Health Observatory framework. Furthermore, Planetary and One Health Literacy could be included as an indicator for the ‘Lancet Countdown on Climate Change & Health’ and for assessing climate-smart health workforce in line with the operational framework for building climate-resilient and low carbon health systems.

Third, the role of Planetary and One Health Literacy needs to be understood from an organizational and systemic perspective (Sørensen *et al*. 2021), such as green healthcare (Mirow *et al*. 2024), so that Planetary and One Health Literacy is woven into the governance and policy processes across different sectors (e.g. health in all policies). Hospitals, health authorities, and universities can act as living laboratories by embedding Planetary and One Health Literacy into their organization development, healthcare strategies, and intersectoral decision-making processes. Furthermore, the Planetary and One Health Literacy perspective needs to be integrated in agendas and actions of different sectors and organizational levels ranging from academia, industry, and civil society to local and international governance, amplifying the targeted impact. Care should be taken to align narratives with indigenous and traditional knowledge and standards across the globe.

These action priorities apply to distinct stakeholder groups. We propose four priority audiences: (i) health educators to integrate Planetary and One Health Literacy into existing curricula and develop training modules for healthcare workers; (ii) policy-makers to adopt literacy indicators into climate-health strategies and health system resilience frameworks; (3) public health institutions to embed these literacies into communication strategies for health promotion and community engagement; and (4) researchers to develop and validate measurement tools and analyse the links between these literacies and behaviour or health outcomes. The research did not require ethical approval because of dealing with theoretical concepts only.

Concluding, investment in Planetary and One Health Literacy through a public health lens is a much-needed game changer to empower people and enhance the health and well-being as part of a systemic response to the planetary polycrisis at hand. This is a call for action across three key areas: (i) embedding Planetary and One Health Literacy into existing education and professional training structures; (ii) advancing targeted research to develop, test, and apply measurable indicators for Planetary and One Health Literacy; and (iii) integrating Planetary and One Health Literacy into organizational governance and policy frameworks and fostering cross-sectoral collaboration. By embracing Planetary and One Health Literacy as a guiding future vision, societies can enable informed decision-making, promote health and equity, and contribute to well-being for all life on Earth.

## Data Availability

Not applicable.
